# Dietary Quality Maintained among Overweight Brazilian Women Enrolled in a Primary Healthcare Service

**Published:** 2014-12

**Authors:** Paula Martins Horta, Aline Cristine Souza Lopes, Luana Caroline dos Santos

**Affiliations:** Federal University of Minas Gerais, Professor Alfredo Balena Avenue, 190 3rd Floor, Room 324, Santa Efigênia, 30130-100, Belo Horizonte, MG, Brazil

**Keywords:** Diet, Diet surveys, Epidemiology, Primary healthcare, Brazil

## Abstract

The present study aimed to evaluate the dietary quality maintained among 113 overweight [body mass index (BMI) ≥25.0 kg/m^2^] women aged ≥20 years, who were enrolled in a Brazilian primary healthcare service in 2009. Dietary quality was evaluated using the Healthy Eating Index (HEI)-1995, which was adapted in the Brazilian context. Statistical analysis included linear regression adjusted by self-reporting energy intake. The prevalence of obesity (BMI ≥30.0 kg/m^2^), elevated waist-circumference, and excessive body-fat were 85.8%, 98.2%, and 100% respectively. Data on dietary quality indicated an HEI score of 66.6 (11.3), with low mean scores for ‘milk and dairy products’ (2.6) and ‘vegetables’ (3.1). The calcium (ß=0.40) and vitamin C (ß=0.27) intake was positively associated with the HEI score. Fat (ß=−0.38) and sodium (ß=−0.21) intake and protein adequacy (ß=−18.17) were inversely associated with the dietary quality. We found that the dietary quality in this study population needs improvement, demonstrating the importance of nutritional counselling within the primary healthcare service.

## INTRODUCTION

Food consumption assessment is essential for understanding the relationship between diet and morbidity/mortality events and for providing information about the determinants of food intake (1), which, in turn, leads to the identification of population groups at risk, making it subsequently possible to propose public health policies or programmes (2). The assessment of food consumption and its associated factors in primary healthcare is essential because it is the preferred gateway to the healthcare system (3).

Studies indicate that populations served by public healthcare services have a high prevalence of inadequate dietary patterns (3,4), which are associated with non-communicable diseases (5), especially overweight and obesity. Thus, the development of valid and useful tools that relate food intake to the incidence of these diseases is of interest (5).

With this in mind, studying diets with various food combinations is more frequently recommended than intake assessment for individual food items or nutrient contribution as food is not consumed in isolation and reflects the choice of a certain lifestyle (6). In this context, the Healthy Eating Index (HEI) assesses the overall intake of food and nutrients and, simultaneously, includes different aspects of the diet (5). Thus, this study aimed to evaluate the dietary quality maintained among overweight women enrolled in a primary healthcare service in Brazil. In this country, the scientific research output on nutrition in primary healthcare is mainly related to the evaluation of nutritional status of children. Investigations on food consumption represent only 12.8% of the bibliography (7).

## MATERIALS AND METHODS

### Study design and sample

We conducted a cross-sectional study among overweight women enrolled in a primary healthcare service in Belo Horizonte, Brazil.

Participants of the study included women who underwent nutritional care in 2009. The inclusion criterion adopted in the study was: being overweight as defined by a body mass index (BMI) of ≥25.0 kg/m^2^ (8). Women who refused to participate in the study, those who were pregnant or lactating, and those who had undergone bariatric surgery were excluded from the study. It should be noted that all the women were physically active and did exercise 3 times a week in a public health promotion service.

The minimum size of the sample was established to be 91 individuals, considering 12.2 as the standard deviation (SD) for the HEI score, 5% as the significance level (α=0.05), and 2.5% as maximum estimative error (9). The SD defined for the HEI score was based on 63.1 (12.2) reported by Boynton *et al*. (6) for overweight and obese post-menopausal women (n=126) in Seattle, WA, USA.

### Data collection

Data were collected by administering a pretested questionnaire (10) during the initial visit of the women for the primary healthcare service. Interviewers were previously trained and consisted of nutrition students of the Federal University of Minas Gerais.

Sociodemographic and economic variables, such as age, occupation, number of residents per household, per-capita income, and educational level, were recorded. In addition, health profile (self-reported morbidity), anthropometric data, body composition, and food consumption were investigated.

Anthropometric evaluation involved measuring weight, height, and waist-circumference (WC) according to the previously-described techniques (8). Nutritional status was classified according to BMI (8) and WC (11).

Body composition was assessed using the bioimpedance method. Recommendations contained in protocols proposed by Kyle *et al*. (12) were adopted when measuring this variable. Ranking of the percentage of body-fat followed the criteria defined by Lohman (13).

A 24-hour dietary recall (R24) was used for determining food consumption. Although performed in a single measure, data collection covered different days of the week and all seasons of the year for the whole sample. Household measurements were used in order to help the participants in reporting the amount of food consumption. Chemical composition of food was analyzed using the software DietWin Nutrition Professional (2008), with the addition of specific Brazilian food composition tables and nutritional information labels of processed foods. In addition, recipes were broken down according to their ingredients to better classify foods in their respective food groups. Data obtained in this analysis enabled the assessment of energy intake and nutrient adequacy according to the principles of the Institute of Medicine (IOM) (14,15) on energy, fibre, macronutrients, and micronutrients (calcium, zinc, iron, sodium, and vitamin A, E, C, D, and B_12_). Consumption of fat fractions was evaluated by recommendation of World Health Organization (WHO) (16).

Dietary quality was evaluated using the HEI devised by Kennedy *et al*. (17), which was adapted for local needs by Fisberg *et al*. (18). The HEI was modified by including the pulse group as an alternative to the saturated fat component because beans are habitually consumed in Brazil. This index is derived from scores distributed across 10 components that characterize different aspects of a healthful diet (18). Each component is evaluated, which scored from 0 to 10, and intermediate values represent the proportion of each component in the intake. The first 6 components of this index include food groups, total fat content, cholesterol level, and sodium level form the other 3 components; and food variety represents the last component (18).

### Statistical analysis

The statistical analysis included simple and multivariate linear regression models to determine the nutritional factors (anthropometry and food consumption) associated with the HEI score. Quantitative variables were transformed in ranks in the case of non-adherence to the normal distribution. All the variables were eligible for the multivariate analysis, independent of the significance level obtained at the univariate level and were entered into the models according to the stepwise criteria. Multiple analyses were adjusted by reporting energy, obtained by a comparison between total energy intake and basal metabolic rate (BMR) (19). BMR was calculated using formulae proposed for women by the IOM according to age, weight, and height (14). Underreporters and overreporters indicated that their energy intake was <1.35×BMR and >2.4×BMR respectively (19).

The data on women whose mean daily energy intake was <832.24 kcal or >4,580.36 kcal—the first and the 99th energy intake percentiles respectively—were excluded from the analysis as recommended by Nielsen and Adair (20).

A 5% significance level (p<0.05) was adopted. Results were presented as mean (SD) for variables with normal distribution and as median [95% confidence interval (CI)] for the other variables. For the statistical analyses, we used Statistical Analysis System software (version 8.02) (1999-2001, SAS Institute Inc., Cary, NC, USA).

### Ethical aspects

This study was conducted according to the guidelines of the Declaration of Helsinki, and all the procedures involving human subjects were approved by the Research Ethics Committee of the Federal University of Minas Gerais. Written informed consent was obtained from all the subjects.

## RESULTS

The study sample included 113 women, with a mean (SD) age of 51.7 (11.1) years, of whom 71.3% were adults (20-60 years old). The monthly per-capita income was US$ 205.0 (95% CI 210.9-263.2), and 51.3% of the subjects were housewife (Table 1).

Regarding the non-communicable disease profile, we found a prevalence of 53.1% for hypertension, 43.7% for dyslipidaemia, and 16.1% for diabetes mellitus (data not shown).

Data on anthropometry and body composition, in turn, indicated the obesity prevalence to be 85.8% (BMI ≥30 kg/m^2^) (8). We also observed a risk rate of 98.2% for metabolic complications according to the WC (very high risk at 85%) (11). The percentage of body-fat indicated a risk rate of 100% for diseases associated with obesity (13) (data not shown).

In relation to the respondents’ energy and nutrient intake, an insufficient energy intake was found in 47.8% of the women, which contrasted with the excessive consumption of fat (22.1%) and its saturated fraction (24.8%). Among the micronutrients, calcium and vitamin D were highly inadequate in 90.3% and 100% of the subjects respectively having intake less than the estimated average requirement (Table 2). The analysis of the self-reported energy intake demonstrated that 54% of the women underreported and 3.5% overreported their energy intake (data not shown).

The data concerning dietary quality indicated an HEI score of 66.6 (11.3), with low suitability for the components referring to the ‘milk and dairy products’ and ‘vegetables’ groups (Table 3).

The results from the univariate analysis are presented in Table 4 and 5. Regarding the final regression model, we observed that approximately 47.5 of the HEI score variance was related to the nutritional factors (total R^2^=0.4746), among which calcium and vitamin C intake was positively associated with the score. In contrast, fat, sodium and protein consumption was inversely associated with the quality of diet (Table 6).

## DISCUSSION

The results of the present study indicate a high prevalence of inadequate food intake, reflecting poor dietary quality maintained among the overweight women enrolled in the primary healthcare service. In addition, the HEI was demonstrated to be a valid index for identifying inadequate nutrition in this scenario, correlating with different parameters of food consumption.

Studies on other adult population have also reported HEI scores between 50 and 70, emphasizing the need for changes in the diet of most subjects. Shah *et al*. (21) conducted a study on overweight women (n=125) and reported a mean HEI-2005 score of 51.4 (0.9). Another study using HEI-1995 observed a mean of 63.7 in 15,658 American individuals (52% women), with a mean BMI of 26.5 kg/m^2^ (22). Chen *et al*. (23) examined the dietary quality of 1,526 American adults with chronic diseases, using HEI-2005 and reported a mean HEI score of 53.6 (0.5).

**Table 1.** Sociodemographic condition and economic profiles of overweight women enrolled in a primary healthcare service, Brazil, 2009

Variable	Value
Age (years)	51.7 (SD: 11.1)
Age range (%)—adults (20-60 years)	71.3
Per-capita income (US$)	205.0 (95% CI 210.9-263.2)
Educational level (years)	7.0 (95% CI 6.1-7.5)
Inhabitants per home	3.0 (95% CI 3.2-3.7)
Housewife (%)	51.3
Retired (%)	15.9
Self-employed (%)	6.2
Unemployed (%)	5.3
Other jobs/functions (%)	18.6

**Table 2.** Calorie and nutrient intake by overweight women enrolled in a primary healthcare service, Brazil, 2009

Dietary parameter	Mean (SD)/ Median (95% CI)	Prevalence (%)			
		Insufficient	Adequate		Excessive
Energy (kcal)	2,041.3 (654.7)	47.8	32.7		19.5
Carbohydrates (%)	56.2 (9.3)	12.4	73.5		14.2
Protein (%)	13.6 (13.4-16.2)	19.5	80.5		0.0
Fat (%)	29.6 (7.4)	8.8	69.0		22.1
Saturated fat (%)	8.4 (2.9)	-	75.2		24.8
Monounsaturated fat (%)	7.9 (2.5)	79.6	20.4		-
Polyunsaturated fat (%)	9.9 (3.6)	14.2	40.7		45.1
Cholesterol (mg)	184.3 (118.8)	-	85.8		14.2
		<EAR	≥EAR≤RDA	>RDA≤UL	>UL
Calcium (mg)	433.9 (301.8)	90.3	7.1	2.7	0.0
Zinc (mg)	7.4 (7.9-10.1)	39.8	13.3	46.9	0.0
Iron (mg)	7.3 (3.4)	44.2	38.9	16.8	0.0
Vitamin A (mcg)	471.1 (599.9-1,038.7)	48.7	8.8	39.8	2.7
Vitamin E (mg)	31.3 (12.4)	2.7	0.9	96.5	0.0
Vitamin C (mg)	55.8 (69.9-115.9)	53.1	5.3	41.6	0.0
Vitamin D (mcg)	1.2 (1.3-2.0)	100.0	0.0	0.0	0.0
Vitamin B_12_ (mcg)	0.9 (0.9-3.9)	79.6	2.7	17.7	0.0
		<AI	≥AI≤UL	>UL	
Sodium (g)	3.3 (1.3)	0.9	23.9	75.2	
		<AI		≥AI	
Fibre (g)	21.6 (9.2)	55.4		44.6	

Al=Adequate intake; CI=Confidence interval; EAR=Estimated average requirements; RDA=Recommended dietary allowances; SD=Standard deviation; UL=Upper intake level

**Table 3.** Healthy Eating Index components score among overweight women enrolled in a primary healthcare service, Brazil, 2009

Healthy Eating Index components	Score (mean/median)	SD/CI 95%
Grains	9.6	7.8 to 8.7
Vegetables	3.1	3.5 to 4.7
Fruits	8.7	5.2 to 6.8
Milk and dairy products	2.6	2.8 to 3.9
Meat and eggs	10.0	7.4 to 8.6
Pulses	10.0	7.2 to 8.5
Total fat	10.0	7.8 to 8.8
Sodium	7.2	5.5 to 6.9
Cholesterol	10.0	8.8 to 9.7
Food variety	5.8	2.5

CI=Confidence interval; SD=Standard deviation

**Table 4.** Simple linear regression between Healthy Eating Index score and quantitative variables relating to nutritional status of overweight women enrolled in a primary healthcare service, Brazil, 2009

Variable	ß (SE)	p value	R^2^
Body mass index	0.21 (0.09)	0.029	0.0424
Carbohydrates	0.25 (0.09)	0.008	0.0615
Fat	-0.32 (0.09)	<0.001	0.1023
Monounsaturated fat	-0.32 (0.09)	<0.001	0.1011
Polyunsaturated fat	-0.31 (0.09)	<0.001	0.0997
Cholesterol	0.23 (0.09)	0.016	0.0516
Calcium	0.47 (0.08)	<0.001	0.2203
Iron	0.26 (0.09)	0.006	0.0674
Sodium	-0.17 (0.09)	0.074	0.0287
Vitamin A	0.35 (0.09)	<0.001	0.1227
Vitamin D	0.42 (0.09)	<0.001	0.1623
Vitamin C	0.37 (0.09)	<0.001	0.1416
Vitamin B_12_	0.41 (0.09)	<0.001	0.1665
Energy/Basal metabolic rate	0.26 (0.09)	0.006	0.0655

SE=Standard error

Regarding the data on dietary quality among the Brazilian population, a study conducted among 228 adults aged ≥60 years, who were residents in the south, indicated a mean HEI-2005 score of 66.6 (10.9) (24). In addition, Fisberg *et al*. (18) reported a mean HEI score of 60.4 (95% CI 59.6-61.2) among 3,454 adults aged 20 years and older, who were living in regions of the state of São Paulo.

Among the HEI components, insufficient scores were obtained for ‘vegetables’ and ‘milk and dairy products’ in contrast to a high median score for ‘cholesterol’ and ‘total fat’, ‘pulses’, and ‘meat and eggs.’ The study conducted in São Paulo reported a mean score of 5.0 for ‘vegetables’ and 2.3 for ‘milk and dairy products’. The ‘cholesterol’ and ‘pulses’ components scored 10.0 and 9.0 respectively (18). Another study reported that HEI-2005 components relating to fruit, vegetables, and dairy products were low-scored in a sample of 4,448 American adults aged 20 years and older (25).

**Table 5.** Simple linear regression between Healthy Eating Index score and qualitative variables relating to nutritional status of overweight women enrolled in a primary healthcare service, Brazil, 2009

Variable	Category	ß (SE)	p value	R^2^
Waist-circumference classification	Without risk or high risk (ref.) Very high risk	- 20.91 (8.36)	0.014	0.0538
Carbohydrate consumption classification	Adequate (ref.) Insufficient Excessive	- -22.38 (9.23) -2.10 (8.72)	0.017 0.810	0.0513
Protein consumption classification	Adequate (ref.) Insufficient	- -19.68 (7.53)	0.010	0.0585
Fat consumption classification	Adequate (ref.) Insufficient Excessive	- -13.88 (10.54) -22.12 (7.22)	0.191 0.003	0.0844

SE=Standard error

**Table 6.** Multiple linear regression between Healthy Eating Index score and variables relating to nutritional status of overweight women enrolled in a primary healthcare service, Brazil, 2009

Variable	ß (SE)	p value	Partial-R^2^
Calcium	0.40 (0.09)	<0.001	0.1761
Fat	-0.38 (0.07)	<0.001	0.1348
Vitamin C	0.27 (0.07)	<0.001	0.0702
Protein consumption classification	-18.17 (5.76)	0.002	0.0531
Sodium	-0.21 (0.07)	0.005	0.0404

SE=Standard error; Total-R^2^ = 0.4746. Analysis was adjusted by energy report

In addition, this study demonstrated that the HEI indicates food intake inadequacies. One method of assessing whether HEI scores represent dietary quality is to link this score to the food and nutrient consumption that are part of it as well as to the consumption of nutrients that are not part of the score itself but are considered integral to a good diet (17). Accordingly, we observed a direct relationship between the HEI score and the calcium and vitamin C intake and an inverse association of this score with total fat, sodium, and protein consumption. In this sense, the women with insufficient protein intake were more likely to have poor dietary quality.

Drewnowski *et al*. (26) who conducted a study with French adults (n=5,081) aged 35–61 years found that higher HEI scores were associated most strongly with higher intake of ‘fruits’, ‘vegetables’, and ‘dairy products’, and with greater ‘variety.’ High HEI scores were also associated with high fibre, beta-carotene, folate, calcium, and vitamin C consumption.

Another study examined the associations between fast-food consumption and dietary quality as measured by the Alternative Healthy Eating Index in 5,633 American adults and elderly (aged 45-84 years). In agreement to the relationship between HEI score and total fat and sodium consumption identified in the present study, we verified that individuals reporting never eating or consuming fast food less than once per week had higher odds of having a healthful diet than those eating fast food once or more than once per week (27).

Finally, Hann *et al*. (28) examined a group of women (n=340) between 21 and 80 years of age and found that the HEI score was associated with dietary variety (*r*=0.71), higher intake of fruits (*r*=0.57), lower total fat intake (*r*=−0.58), saturated fat fraction (*r*=−0.56), and higher plasma concentration of alpha- (*r*=0.40) and beta-carotene (*r*=0.28) and vitamin C (*r*=0.26), among others.

### Limitations

The limitations of this study include its cross-sectional design which does not enable causality comprehension and food intake assessment in just 1 day, which might not represent habitual intake (29). In addition, the study was limited because it had a high prevalence of underreported energy intake and because variables relating to household food security and women's knowledge on food were not considered.

### Conclusions

Evaluating dietary patterns helps in identifying population groups at risk, which may be useful in making proposals for public health policies or programmes. Our findings indicate poor dietary quality among the women enrolled in the primary healthcare service in Brazil. Moreover, HEI has proven to be a valid index for inadequate nutrition in the present scenario. Application of other dietary indices and conducting further studies on primary healthcare services are important steps in characterizing food patterns in this context.
